# From Physics to Bioengineering: Microbial Cultivation Process Design and Feeding Rate Control Based on Relative Entropy Using Nuisance Time

**DOI:** 10.3390/e20100779

**Published:** 2018-10-11

**Authors:** Renaldas Urniezius, Vytautas Galvanauskas, Arnas Survyla, Rimvydas Simutis, Donatas Levisauskas

**Affiliations:** Department of Automation, Kaunas University of Technology, Kaunas LT-51367, Lithuania

**Keywords:** gray box, relative entropy, microbial cultivation, numerical convex optimization, parsimony, nuisance time

## Abstract

For historic reasons, industrial knowledge of reproducibility and restrictions imposed by regulations, open-loop feeding control approaches dominate in industrial fed-batch cultivation processes. In this study, a generic gray box biomass modeling procedure uses relative entropy as a key to approach the posterior similarly to how prior distribution approaches the posterior distribution by the multivariate path of Lagrange multipliers, for which a description of a nuisance time is introduced. The ultimate purpose of this study was to develop a numerical semi-global convex optimization procedure that is dedicated to the calculation of feeding rate time profiles during the fed-batch cultivation processes. The proposed numerical semi-global convex optimization of relative entropy is neither restricted to the gray box model nor to the bioengineering application. From the bioengineering application perspective, the proposed bioprocess design technique has benefits for both the regular feed-forward control and the advanced adaptive control systems, in which the model for biomass growth prediction is compulsory. After identification of the gray box model parameters, the options and alternatives in controllable industrial biotechnological processes are described. The main aim of this work is to achieve high reproducibility, controllability, and desired process performance. Glucose concentration measurements, which were used for the development of the model, become unnecessary for the development of the desired microbial cultivation process.

## 1. Introduction

Theoretical aspects for the practical application of adaptive control systems that operate in unknown, nonlinear, and time-varying biotechnological environments are still to be developed, investigated and implemented. For historic reasons, and reproducibility and restrictions imposed by regulations, the open-loop control approach dominates in industrial cultivation processes. The scope of industrial challenges, when dealing with adaptive control, consists of but is not limited to adequate (sufficient) modeling methods, the lack of direct measurements of the state variables and their indirect estimation tools, and the reliability of sensors.

To speed up the implementation of advanced control systems for the control of biotechnological processes the USA Food and Drug Administration (FDA) has announced a well-known Process Analytical Technology (PAT) Initiative [1]. One of the central topics of this initiative is the requirement to accelerate the elaboration and implementation of advanced control systems in the biotechnological industry. The FDA have stated that “the goal of PAT is to understand and control the manufacturing processes, which is consistent with our current drug quality system: *quality cannot be tested into products, it should be built-in* *or should be by design*” [1].

Feeding of glucose solution serves as the main limiting factor to assure the controllability of an aerobic cultivation of Escherichia coli [2,3]. The purpose of controllability is to ensure the conditions by which the control action (substrate feed rate) allows the control of the growth rate and avoids substrate inhibition on biomass growth and/or phase conversions to metabolisms which lead to the formation of inhibiting products, e.g., acetates [4,5,6]. Efficient substrate feeding strategies should ensure controllability of the process and secure sufficient bioprocess productivity [7]. Depending on the particular bioprocess, productivity is considered as the total amount of biomass [8] or target product accumulated at the end of the cultivation process, e.g., the target is a recombinant protein [9,10].

Recent modeling approaches of varying complexity have aimed to speed up the development of biotechnological processes [6,11,12,13,14,15]. The established bioprocess model not only allows the process behavior to be forecasted and its performance to be increased with feedback control [3], but it also increases its reproducibility [16] and makes model-based verification possible [17]. Another advantage of bioprocess modeling is that it enables the indirect estimation of the bioprocess state and helps to produce feedback control on the cultivation system variables based on fundamental knowledge. One example of this is controlling the level of dissolved oxygen *pO*_2_ [18]. This avoids excess feedback control when disturbances in sensors occur, e.g., during the addition of antifoam to a bioreactor.

The development of desired bioprocess should involve designing a feeding rate time profile by relying on Occam’s razor [19]. Consequently, the motivation is that the gray box model [20] should represent basic regularities of microbial cultivation processes. The behavior of the biomass growth model should reflect the majority (to follow the Pareto principle) of industrial cultivation processes. The main assumption of the current work is that 80% of cultivation process variation depends on a substrate feeding rate and effectively on substrate concentration in the cultivation medium.

After choosing the gray box model to be used for fitting and smoothing the glucose consumption data, the authors’ choice is to apply the maximum (relative) entropy (ME) as a method to update from the prior probability distribution to the posterior distribution [21]. A similar choice by Caticha and Preuss in 2003 followed their successful applications of ME in statistical mechanics. In 2007, Giffin and Caticha [22] showed that a Bayesian updating approach is a special case of the maximization of entropy, and this idea later worked as catalyst to develop applications in other fields of science, including, but not limited to, information flows in dynamic [23] and complex systems [24], new inspirations in frameworks analyzing the bounds of estimation error variances for a general state estimation system [25], or a tool for generating random bits in microprocessors dedicated for bio-signals [26], etc. A recent review [27] contains further references describing the path from communication systems (information theory) to control theory.

In this study, Gaussian prior distribution represents the data noise but with the additional assumption that the model’s uncertainty is equal to the square of the model’s local mean. This idea originates from an optimal resource allocation study by Renaldas Urniezius (R.U.) [28] where the deviation of bank remittances to a common ATM network warehouses was proportional to the bank’s market share. Thus, over time such a technique “by-design” forced banks to follow a “fair game” rule.

The current work’s convex optimization routines’ analogy to statistical mechanics is important: Lagrange multipliers and their estimation procedures are treated similarly to how convex optimization routines using ME criterion are implemented in model fitting problems. In 2013, R.U., in cooperation with Adom Giffin, applied the ME numerical convex optimization implementation. That was the first attempt of a generic implementation of any number of boundary constraints for the criterion of the ME on any prior distribution. The effort involved the development of a generic ME package for the *R* project of statistical computing [29]. As a result, upon numerical verification the results matched global extremum numerical solutions found in several public papers, e.g., [30,31]. Later in this paper, the description of a nuisance time is used for the purpose of explaining the implementation of numerical convex optimization.

A study of optimal resource allocation [28] introduced a numerical semi-global optimization approach which is relevant to other models (although not necessarily restricted to the models of econometrics). This work is an example of how ME helps to develop high-speed numerical semi-global optimization routines for a multivariate problem of gray box model fitting. This study represents the authors’ effort to develop substrate solution feeding profile in bioengineering.

The implementation of numerical semi-global optimization routines is analyzed simultaneously, paying attention to enforcing initial and boundary conditions. Practical benefits in new industrial process design are also highlighted. Finally, options and alternatives are discussed, which help to achieve high reproducibility, controllability, and desired process performance. The software tool and its graphical user interface, which were developed for this purpose, are also introduced in this paper.

In this paper, Section 2 lays out the workflow of biomass growth model identification. First, the maximum substrate consumption profile is found by performing fed-batch cultivation with feeding carried out in portions; the next subsection describes the motivation for gray box model selection and its probabilistic assumptions; the third subsection contains the derivation of the optimization criterion for model fitting; the fourth subsection exposes the relationship of this study’s numerical routines to the convex pathway, which is inherited from physical applications; and the fifth subsection describes the implementation of the numerical algorithm to identify the gray box model parameters. The third section presents a flexible software tool which was developed for the generation of limited biomass growth substrate feeding profiles. The fourth section consists of experimental analysis: a practical illustration of bioprocess reproducibility and controllability achievement; an assessment of goodness of fit to dataset, acquired from a third party; and sensitivity analysis of the numerical routines to the seed values of initial parameters, which shows the practically beneficial outcome of the convex outlook in this work.

## 2. Identification of Biomass Growth Model 

Prior to the calculation of the substrate feeding rate time profile, the time profile of the maximum glucose consumption rate must be identified. The obtained maximum glucose consumption rate, under controlled penetration into the area of aerobic fermentation, is the most important parameter for determining the maximal available specific growth rate μ during different cultivation phases, where μ is estimated from:

(1) $$\mu =\frac{dX}{dt}\cdot \frac{1}{X}$$

where *X* is the total biomass. The specific growth rate μ is the most important precondition to ensure process controllability [32]. By choosing a different limitation level the desired performance of the controlled process can be realized. There are several ways to approach this task. Here, a classical straightforward procedure helps to identify the maximal oxidative capacity of cells, assuming that during whole cultivation process:

- the biomass yield on substrate (i.e., $Y_{X/S}=\frac{dX}{dS}$ [g/g], where S represents the substrate) does not change.
- limited feeding rate will ensure avoidance or changes in metabolic pathways.

The time profile of the maximum rate of substrate consumption can be determined from fed-batch processes with feeding carried out in portions, which represent substrate dosing oscillation cycles [32]. At each oscillation cycle glucose concentration is measured. If it falls below the threshold, additional substrate solution is added so that the glucose concentration reaches the desired level. The change of the concentration’s decrease matches its actual consumption throughout the time discretization interval. Another alternative to estimate glucose consumption is indirect calculation from the optical density (OD) measurements.

After the substrate consumption data is known, the selected gray box model is fitted to the experimental data. This model contains a partial form of the theoretical information about microbial strain growth properties and additional nuisance/perturbation dimensionless parameters. In the beginning of the process, exponential growth is assumed. This can be explained by the fact that, in the beginning of the process, specific biomass growth rate usually increases or remains constant. After the exponential phase of a microbial cultivation, the induction (e.g., with isopropyl β-d-1-thiogalactopyranoside (IPTG)) is performed [33]. After the induction, the specific growth rate decreases; for a description of this phenomenon, the bioprocess model includes the nuisance parameter for the linear decline of the specific growth rate over time. Both stages have the same first- and second-order boundary condition to guarantee their smooth intersection by model fitting procedures during ME optimization.

### 2.1. Fed-Batch Cultivation to Identify the Maximum Substrate Consumption Profile

The first step of process design is usually to estimate the maximal available specific growth rate at different process phases. For this purpose, a common approach is to apply fed-batch cultivation with dosed glucose feeding, i.e., to obtain both the glucose cumulative consumption data and high biomass yield, fed-batch cultivation with feeding carried out in portions is a common choice [32]. At the start of each episodic oscillation cycle, depending on the output of glucose concentration measurements, the process involves periodic substrate refilling so that the glucose concentration reaches the desired level. Normally, this level is selected so as to be high enough to still anticipate some of the glucose concentration at the end of the step. Both a cultivation medium volume in the bioreactor, and the glucose consumption, are recalculated at each oscillation cycle, which usually lasts around 30 min to 1 h. There are at least two practical choices to retrieve glucose consumption for every oscillation cycle:

1. Measuring glucose concentration prior to each cycle and after it finishes. Prior to each cycle, immediately after the glucose concentration has reached the desired concentration due to substrate addition, the glucose consumption is calculated during the most recent step by subtracting the multiplication of the cultivation medium volume in the bioreactor and glucose concentration, at the start of the interval, from the multiplication of the cultivation medium volume in the bioreactor and glucose concentration at the end of the interval, immediately before the next substrate prefill.
2. The second alternative is based on OD observations. To retrieve a cumulative glucose consumption during each oscillation cycle, the OD value (in o.u.) is multiplied by both a coefficient of biomass concentration (approximately 0.4 g/L/o.u.) and cultivation medium volume in the bioreactor, and is then divided by a biomass yield of glucose (approximately 0.5 g/g) [34]. Subtraction of the cumulative glucose weight at the start of the oscillation cycle from the one at the end of the same cycle gives the total glucose consumed during the step.

Choosing between glucose consumption calculation techniques depends on practical considerations. Usually, due to the bioprocess lag phase immediately after inoculation to a bioreactor, glucose concentration measurements are skipped. This happens at the beginning of a bioprocess. The main reason for this is that it is sufficient to monitor the dynamics of a dissolved oxygen level to decide whether the substrate feeding control must be started [35]. In such cases, the glucose consumption can be estimated from OD measurements as described in the second alternative above. However, after the lag phase of a cultivation, when the glucose concentration measurements are started, the first alternative becomes a better solution.

Until this point, the procedure consisted of the calculation of the glucose consumption profile in liters per hour. The feeding substrate solution has a certain density and glucose concentration in the solution. Therefore, the retrieval of the substrate solution weights still requires multiplication of the current profile by substrate density value (g/L) and its division by the glucose concentration of the feeding substrate. This finally yields the feeding solution profile to be processed by an industrial controller with weighing scales attached. This profile represents the maximum oxidation capacity, which does not necessarily mean the maximum expression of a product. Therefore, the design of the glucose feeding rate time profile would help to avoid glucose overfeeding and the metabolism pathways associated with it. For this purpose, a time dependent model is necessary to effectively fit the feeding profile data.

### 2.2. Gray Box Model Selection and Probabilistics Assumptions

Time dependence is one of the key properties of the “black box” model to assure that slowing down the time variable with respect to actual physical time would help to preserve the shape of a feeding profile. A parametric construction of model structure makes this model a gray one since some of its structure is inherited from fundamental knowledge about the bioprocess. In other words, the basic course of the process can be described by a family of exponential relationships, and this partially matches fundamental principles of cell biomass growth. The whole bioprocess consists of two stages, and may be described by two gray box models. The first one covers both lag and exponential stages of the cultivation process:

(2) $$x_{t<t_{induction}}\left(t\right)=k_{11}\cdot e^{k_{12}t}-k_{13}\cdot t+k_{14},$$

where x(t) is a one-dimensional variable containing the cumulative amount of feeding substrate to be fed during the first stage at time t, $t_{induction}$ is a time stamp of an induction moment (or a time close to it, since the match is a soft requirement) and coefficients $k_{1i}$ are the gray box model’s nuisance parameters for the first stage of the process. The undetermined character of the gray box model will be discussed later. The exponential growth rate is a first-order reaction and is approximately linearly proportional to the concentration of a biomass or the total cell number at a constant specific growth rate (in case the substrate and other factors do not inhibit the specific growth [36]). Even if the parameter $k_{12}$, by theoretical exponential growth form, reflects the growth rate, its numerical value is not necessarily equal to the value of μ. This is one of the benefits of ME optimization for such an undetermined system.

The second stage model defines how the process behaves after the time of induction (or after a time close to the induction time stamp):

(3) $$x_{t\geq t_{induction}}\left(t\right)=k_{21}\cdot e^{k_{22}\cdot t+k_{25}\cdot t^{2}}-k_{23}\cdot t+k_{24},$$

where coefficients $k_{2j}$ are the gray box model’s nuisance parameters, identified for the second stage of the process. This second stage model represents the decelerating stage of the bioprocess cultivation. It contains additional parameter $k_{25}$, which reflects the dynamics of deceleration of the specific growth. However, its numerical value does not necessarily match the value of the actual specific growth rate deceleration divided by two. This is the abovementioned benefit of ME for undetermined systems. To reiterate, numerical values of all the gray box model parameters might not necessarily have a physical meaning, however, they still serve as a rational choice for model fitting to be used in later scaling-up or scaling-down of the industrial processes using ME.

The application of the gray box model for time series probabilistic variables [37] in ME first requires the selection and definition of the probabilistic distribution of the likelihood. For this purpose, the gray box model’s expression is generalized to:

(4) $$x_{m}\left(t\right)=k_{m1}\cdot e^{k_{m2}\cdot t+k_{m5}\cdot t^{2}}-k_{m3}\cdot t+k_{m4},$$

where the index m is equal to 1 for the first-stage model and equal to 2 for the second-stage model, and $t_{k}$ is the time when the cumulative glucose consumption $x_{m}\left(t_{k}\right)$ for the *k*-th oscillation cycle has been calculated. Similarly to the procedure in [37], Equation (4) is the constraint over the mean of a probabilistic variable $\langle x_{m,k}\rangle$, which also represents the prediction value of the proposed model:

(5) $$\langle x_{m,k}\rangle \equiv \langle x_{m}\left(t_{k}\right)\rangle =k_{m1}\cdot e^{k_{m2}\cdot t_{k}+k_{m5}\cdot t_{k}^{2}}-k_{m3}\cdot t_{k}+k_{m4},$$

where $k_{15}$ is set to zero by default. Then posterior distribution at *k*-th oscillation cycle is:

(6) $$P_{posterior}\left(x_{k}\right)\sim N\left(\langle x_{m,k}\rangle ,\sigma_{\langle x\rangle }^{2}\right),$$

which assumes that all posterior variances $\sigma_{\langle x\rangle }^{2}$ are the equal.

A probability density function defining the prior distribution is assumed also to be Gaussian, represented by a joint prior [38]:

(7) $$P_{likelihood}\left(x_{k},cx_{m,k}\right)\sim N\left(cx_{m,k},cx_{m,k}^{2}\right),$$

where $cx_{m,k}$ is observation value represented by substrate feeding profile value at the time discretization step *k*.

Equation (7) shows that the mean of each observation (calculated value of cumulative substrate feeding solution) is proportional to its deviation value. An idea of evaluation of the biomass prediction error or bias through the percentage to the actual observed value is not new. Mean absolute percentage error (MAPE) frequently serves as a tool for biomass prediction or estimation accuracy [39,40]. Interestingly, according to the authors’ practical experience with microbial cultivation processes, such an assumption is also critical as a soft constraint for the fitting of substrate feeding solution models. However, such a hypothesis of this numerical phenomenon requires further testing. There are several possible explanations for it:

- The exponential stage is one of the main stages of the whole bioprocess. One of the exponential distribution properties is that its variance matches the squared mean. The same applies to gamma distribution, which is a special case of exponential distribution.
- Measurements of optical densities at higher densities involve increasing measurement errors due to dilution of the samples, which might cause exponentially increasing bias.
- It is normal for optical densities to increase by more than 300 times during the process. Consequently, any local or microscopic measurements, such as glucose concentration and OD measurement, involve increasing bias from the macroscopic point of view.
- From the point of view of classical control theory, there are nonlinear system parts which will influence the feedback, and their effect intensifies as the concentration of biomass increases—for example, the permeability of the feeding hose, any external disturbance on the weighting scale equipment, overpressure changes in bioreactor, etc.

MAPE evaluation has statistical disadvantages (none of which, however, is a major drawback for biomass estimation):

(1) There will never be zero biomass concentration after inoculation into a bioreactor.
(2) The lag phase covers smaller OD values and smaller estimation errors are literally expected during this period.
(3) The maximum OD values usually depend on the strain cultivated, so there are no expected comparison issues when considering the repeatability of the estimator.

The idea of having process variable participate as uncertainty originates from financial applications where a fair game condition, between banks of variable sizes, was achieved by treating the annual bank turnover as the uncertainty for the bank’s remittances to ATM network. This showed both performance and optimum wise satisfactory results [28].

Construction of prior and posterior distributions allows shifting to the actual model fitting.

### 2.3. ME Criterion Derivation

Prior to model fitting, the resolution of expression of relative entropy for a single discrete time moment *k* starts with:

(8) $$S_{k}\left(P_{posterior},P_{likelihood}\right)=-\int_{-\infty }^{\infty }P_{posterior}\left(x_{k}\right)\cdot \ln\frac{P_{posterior}\left(x_{k}\right)}{P_{likelihood}\left(x_{k},cx_{k}\right)}dx_{k}$$

Integration of Equation (8) yields:

(9) $$S_{k}\left(P_{posterior},P_{likelihood}\right)=-\frac{{\left(\langle x_{m,k}\rangle -cx_{m,k}\right)}^{2}}{2\cdot cx_{m,k}^{2}}-\frac{1}{2}+\frac{\sigma_{\langle x\rangle }^{2}}{2\cdot cx_{m,k}^{2}}+\ln\frac{cx_{m,k}}{\sigma_{\langle x\rangle }}+c_{int},$$

where additive terms, integration constant $c_{int}$ and division by a factor of 2 can be omitted, when constructing “cumulative” relative entropy for all steps of a bioprocess:

(10) $$S\left(P_{posterior},P_{likelihood}\right)=-\sum_{ki=1}^{n_{i}}\frac{{\left(\langle x_{1,ki}\rangle -cx_{1,ki}\right)}^{2}}{cx_{1,ki}^{2}}-\sum_{kj=1}^{n_{j}}\frac{{\left(\langle x_{2,kj}\rangle -cx_{2,kj}\right)}^{2}}{cx_{2,kj}^{2}},$$

where $n_{i}$ and $n_{j}$ are the total number of cumulative feeding solution weights prior to and after the induction time, $\langle x_{1,ni}\rangle =\langle x_{2,1}\rangle$, and $cx_{1,ni}=cx_{2,1}$ due to intersection restriction.

There are two pieces of information that have still to be accounted for in the relative entropy: initial and boundary conditions. The initial biomass concentration condition is relevant, because the shake flask procedures, prior to inoculation, are usually standardized in such a way that biomass concentration is known with smaller uncertainty at the beginning. Since $n_{i}+n_{j}$, i.e., the total number of fed-batch steps, is usually less than 50, the reduction of the variance of the first step by a factor of 20 provides satisfactory results as a soft constraint for numerical ME optimization routines.

The main boundary condition of the optimization task is making certain that the model fitting curves originating from Equations (2) and (3) intersect smoothly. The time at which both model curves intersect usually corresponds to the induction time, however this is not a strict requirement. The mismatch sometimes indicates that the bioreactor system failed or was planned improperly to induce the IPTG at the right time. However, such discrepancy might be an expected designed behavior which is subject to the requirements of the process development.

Three soft restrictions on the rate of change of substrate feeding profile eventually all lead to satisfactory model fitting results. The first ensures that both curves should have the same rate of change at the intersection. The uncertainty of this variance is divided by a factor of 2 compared to Equation (10). The most recent substrate feeding value, prior to the intersection, and the first value immediately after it, both conclude the remaining two loose data constraints. However, the variance is multiplied by a factor of 100 this time, because there is more uncertainty introduced by a considerable time difference. It is anticipated that these two last soft constraints improve smoothness from an acceleration perspective at the intersection.

The rate of change of the statistical means Equation (5) has closed form parametric expressions by differentiation:

(11) $$\langle {\overset{´}{x}}_{m,k}\rangle =\left(2\cdot k_{m5}\cdot t_{k}+k_{m2}\right)\cdot k_{m1}\cdot e^{k_{m2}\cdot t_{k}+k_{m5}\cdot t_{k}^{2}}-k_{m3}$$

Thus, the final form of ME Equation (10) criterion becomes:

(12) $$\begin{array}{c} S\left(k_{mi},k_{mj}\right)\equiv S(\langle x_{m,k}\rangle ,\langle {\overset{´}{x}}_{m,k}\rangle )=-\frac{{\left(\langle x_{1,1}\rangle -cx_{1,1}\right)}^{2}}{0.05\cdot cx_{1,1}^{2}}-\overset{n_{i}}{\underset{ki=2}{\sum }}\frac{{\left(\langle x_{1,ki}\rangle -cx_{1,ki}\right)}^{2}}{cx_{1,ki}^{2}}\\ -\overset{n_{j}}{\underset{kj=1}{\sum }}\frac{{\left(\langle x_{2,kj}\rangle -cx_{2,kj}\right)}^{2}}{cx_{2,kj}^{2}}-\frac{{\left(\langle {\overset{´}{x}}_{1,ni}\rangle -\langle {\overset{´}{x}}_{2,1}\rangle \right)}^{2}}{0.5\cdot cx_{2,1}^{2}}-\frac{{\left(\langle {\overset{´}{x}}_{1,ni-1}\rangle -\langle {\overset{´}{x}}_{2,0}\rangle \right)}^{2}}{100\cdot cx_{1,ni-1}^{2}}-\frac{{\left(\langle {\overset{´}{x}}_{1,ni+1}\rangle -\langle {\overset{´}{x}}_{2,2}\rangle \right)}^{2}}{100\cdot cx_{2,2}^{2}}. \end{array}$$

Equation (12) recovers the weighted sum of residuals [41,42] and manifests two benefits of ME. The intuitive manipulation on uncertainty led to the introduction of weighting factors to the optimization routine and made it possible to consider both initial and boundary conditions. Thus, the criterion still preserves its form’s simplicity, circumventing variational Lagrangian or Hamiltonian [43] formulations. As Equation (12) presents the same system as Equation (8), for the purpose of numerical search for the unknowns $k_{mi}$ and $k_{mj}$, the multivariate path can be explored in a computationally efficient way, in the same manner as how Lagrange multipliers lead prior distribution to a posterior one in physics [21,44], which is a topic of the following subsection.

### 2.4. Nuisance Time in Convex Optimization Trajectories

This work’s principle of convex optimization lies in the updating of a prior probability density function to the posterior one by employing:

(13) $$P_{new}\left(\theta \right)=\frac{P_{old}\left(\overset{´}{x},\theta \right)\;e^{\beta \cdot f\left(\theta \right)}}{\int P_{old}\left(\overset{´}{x},\theta \right)\;e^{\beta \cdot f\left(\theta \right)}d\theta },$$

where all notation descriptions are presented in [41]. Equation (13) substitutes the idea of how the prior distribution transforms to the posterior one. If the Lagrange multiplier β is zero, then the posterior distribution recovers its prior knowledge. In other words, a prior function is a special case of a more general posterior one and a scalar value of β (or any parameter(s) of the posterior function) increasing from zero to a certain value defining the transformation “trajectory” between the two distributions. It also ensures both the compliance with constraints and the maximization of entropy. A similar conversion trajectory effect took place in a revisiting of the method of mirror images [45] and vector normalization [46], where a variance approaching zero produced vector normalization expressions, i.e., the spatial point with the Dirichlet boundary condition imposed.

**Figure 1:**
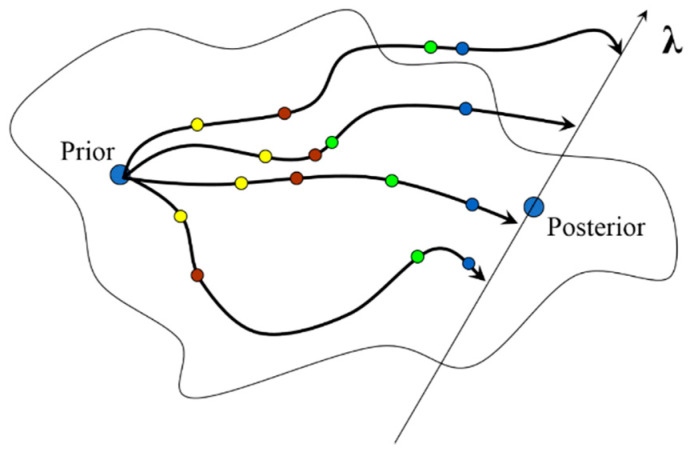
Multivariate path of Lagrange multipliers starting at the state of a prior distribution, where all Lagrange multipliers have zero values, and evolve to a posterior distribution.

Figure 1 depicts the transformation in the Lagrange multipliers configuration space, where the set of Lagrange multipliers starts at zero value of a prior point and ends at the posterior solution axis.

Figure 1 portrays nuisance trajectories for each Lagrange multiplier in ***λ*** configuration space, where circles filled with a color portray the states in a pseudo time, which is defined as a *nuisance time*. These states are the optimization stages of the entropy maximization, in case of sampling it while updating from prior to posterior function. The meaning of nuisance time and nuisance trajectories is similar to that of nuisance parameters [47], and these must be accounted for. Despite the fact that there might be no interest in intermediate information when updating from prior to posterior distribution, nuisance trajectory is important. The main reason for this is that in many cases there are no closed-form solutions for such a transition, so iterative tactics remain the only choice, i.e., as in the generic implementation of ME optimization for any set of constraints.

*The nuisance time and its trajectories define, from an algorithmic point of view, how numerical convex optimization must be implemented*.

Figure 1 shows that any subsequent step, receding from the prior information, must follow the trajectories which all maximize the convex criterion. This implies that any progress towards posterior function must be made simultaneously for all nuisance parameters. In other words, a set of changes for all parameters should be chosen such that the next advance made maximizes the convex criterion.

Similarity of the nuisance time and its implications exists to the parsimony principle [48], where a parsimonious model is the “one, in which many of the entries of the parameter matrices are zero” [49]. By comparison, with the nuisance time, the numerical convex optimization starts with all zeros, i.e., banks are frugal, unwilling to use resources, and prefer not to remit their portion [28] or a posterior distribution stays in the form of a prior function, if no updating is performed or constraints imposed [38]. Moreover, the nuisance time and parameter trajectories play similar roles when parameters are dependent on other functions, as adjoint variables are dependent on time in Hamiltonian formulations of Pontryagin’s maximum principle [50]. If the values of adjoint variables contain zero values for any time, the constraints to which these adjoint variables are assigned to have no effect on the Hamiltonian output.

A trade-off between goodness of fit [51] and parsimony is resolved by the undetermined gray box model formulation, which potentially has an infinite number of local extrema. The interesting fact is that there is no major distinction between the goodness of fit properties at these extrema, so the one which leads to the solution by following the convex pathway is chosen as the rational choice. The investigation of physical nuisance parameter values is out of interest in this study, and their projections to the coordinate space of compiler’s variable types is an acceptable supposition to achieve the goal for model fitting. By comparison, the authors of [48] state that “if a model has many free parameters—for instance, a complex budget constraint or complex household preferences—then the model is relatively nonparsimonious”. Such a statement is not relevant, and overfitting issues [52] do not occur in the scope of ME optimization for the profile fitting. On the contrary, both parsimony and multiple parameters helped to arrive at a competitive local extremum, which has equivalent goodness of fit compared to others extremum candidates. Moreover, the gray box model itself inherits some of the known theoretical structure and the convex pathway through nuisance time and trajectories produces local extremum choices that result from ME.

### 2.5. Identification of the Gray Box Model Parameters Using Nuisance Time

**Figure 2:**
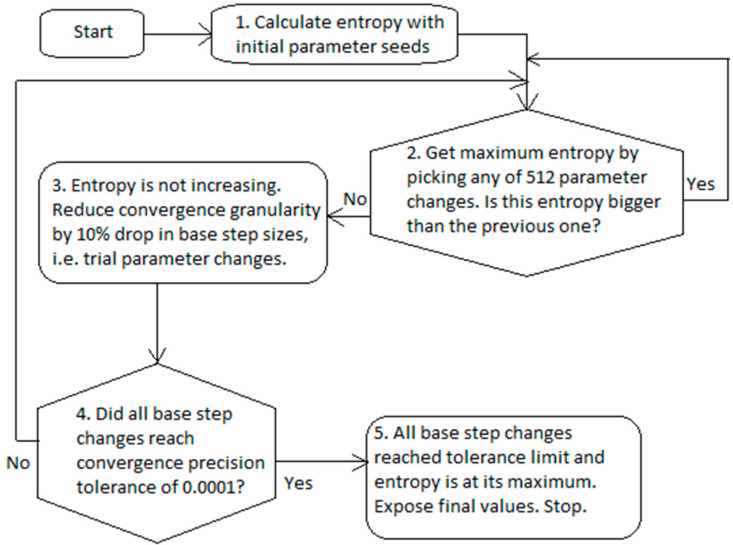
The workflow of numerical building blocks for identification of the gray box model.

The workflow of numerical identification of the gray box model parameters consists of two proactive parts (Figure 2). The first part involves choosing the best unique parameter change, which maximizes entropy. The second part involves reducing the parameter progress when the relative entropy is no longer increasing.

The entropy calculation of numerical blocks #1 and #2 (Figure 2) uses Equation (12). The identification of gray box parameters in numerical block #2 (Figure 2) uses both Equations (3) and (4) and consists of two major steps:

1. For historic reasons, the resolution of $k_{m1},k_{m3},k_{m4}$ is performed by a linear regression through the maximization of the following measure:

(14) $$S\left(k_{m1},k_{m3},k_{m4}\right)=-\sum_{ki=1}^{n_{i}}{\left(\langle x_{1,ki}\rangle -cx_{1,ki}\right)}^{2}-\sum_{kj=1}^{n_{j}}{\left(\langle x_{2,kj}\rangle -cx_{2,kj}\right)}^{2},$$

This ends with the closed-form $k_{m1},k_{m3},k_{m4}$ expressions, with the only exception being that $k_{14}:=-k_{11}$ due to the preference to keep the estimate of the initial magnitude of the profile between zero and the value of the first calculated substrate feeding. The values of $k_{12},k_{22},k_{25}$ are passed from the second step, which encloses this step; and

2. This step is the major convex optimization step which follows the trajectories of nuisance time and deals mainly with the determination of $k_{12},k_{22},k_{25}$ parameters by following the maximum entropy direction, which is elaborated below.

At the beginning, numerical block #1 in Figure 2, the gray box model in Equation (12) is parsimonious, i.e., all $k_{12},k_{22},k_{25}$ values are set to zero, the first step is executed and initial entropy using Equation (12) is acquired. Then, in numerical block #2 of Figure 2, all combinations of $k_{12},k_{22},k_{25}$ are checked by adding to each of them $step_{12},step_{22},step_{25}$ step sizes originating from the following candidate collection: −6.4, −1.6, −0.4, −0.1, 0.1, 0.4, 1.6, and 6.4. Thus, entropy Equation construction of the step size 12 is calculated in total for 512 combinations between the all three parameters. The rule of trial collection is the following:

- At the start of program execution, the initial base step sizes are $basestep_{12}:=0.1,basestep_{22}:=0.1,basestep_{25}:=0.1$ for all three parameters.
- Then, $\pm basestep_{12},\;\pm 4\cdot basestep_{12},\;\pm 4^{2}\cdot basestep_{12},\;\pm 4^{3}\cdot basestep_{12}$ all compose the step collection for $step_{12}$, and similarly candidate enumerations are formed for both $step_{22}$ and $step_{25}$.

There are other sampling window strategies which depend on the threshold dedicated for relative entropy [53], however, the authors’ rational choice was to keep sampling sparse enough for the model fitting purposes and postpone investigating sophisticated approaches for a future. After all 512 combinations of $step_{12},step_{22},step_{25}$ are tested for their entropies, the best one is chosen, i.e., the one which has the maximum entropy compared to the other candidates, and the program permanently updates the search parameters by:

(15) $$k_{12}:=k_{12}+step_{12},k_{22}:=k_{22}+step_{22},k_{25}:=k_{25}+step_{25}$$

This revision procedure of $k_{12},k_{22},k_{25}$ is repeated until the maximum entropy stops increasing. This means that the program execution most likely approached the region of the local extremum and step granularity became too sparse to converge further. Then, the program decreases the base step sizes as in numerical block #3 of Figure 2 as follows:

(16) $$basestep_{12}:=basestep_{12}/1.1,\;basestep_{22}:=basestep_{22}/1.1,\;basestep_{25}:=basestep_{25}/1.1,$$

and the program again proceeds with the same construction of a trial collection and entropy maximization as earlier.

When three base steps all reach a convergence precision tolerance of lower than 0.0001 (numerical block #4 in Figure 2), the parameters converged to the optimal solution. Finally, several safeguards are put into effect against abnormal numerical scenarios, which return minimum variable type value, so that these cases do not interfere with the maximization of entropy. These safety measures mainly reject a candidate collection, if a solution for Equation (14) does not exist for that set, e.g., omitting cases with infinitesimal solutions or divisions by zero.

In conclusion, a nuisance time implicitly plays a significant role in this algorithm. After each ME iteration, when the new maximum entropy value is acquired, the program returns a set of so far optimal combinations of the gray box model parameters. This interim set of parameters belongs to the same nuisance time, so the program knows exactly which parameters progress faster than others in the nuisance time coordinate system. A software progress bar would reflect this effect of a numerical optimization. Thus, the program follows the convex pathway of the whole functional as with the Lagrange multipliers and update the prior distribution to the posterior one—interestingly by going along the maximum relative entropy pathway, which supposedly is a rational choice as well.

After the optimal gray box model parameters are known, the software tool’s graphical user interface allows manipulation by various options of the profile shape, which can later help in attempting to maximize a product or seek a better target protein expression.

## 3. Bio-Engineering Software Tool for the Design of Feeding Rate Time Profiles and the Development of Cultivation Processes

**Figure 3:**
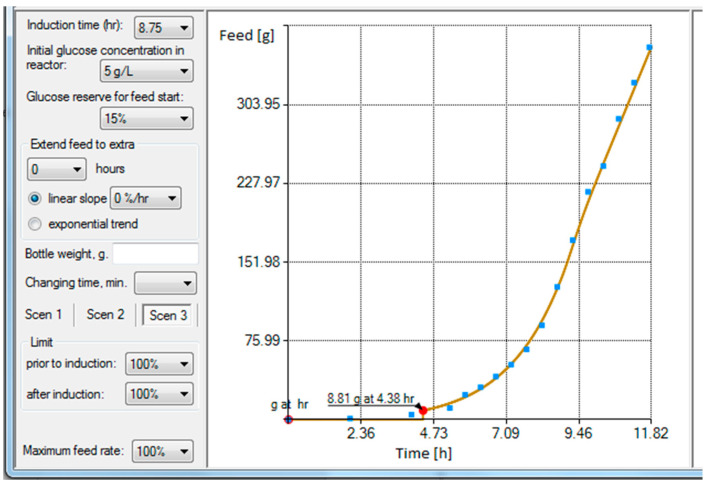
Substrate feeding rate profile designer with engineering options and cumulative profile view from the left side of the operator dialog screen.

The development process of achieving reproducibility, controllability, and desired process performance requires not only the feeding profile identification representing the maximal oxidation capacity of a microbial culture, but also a flexible engineering tool (Figure 3) to change how the feeding profile is reshaped in order to gain high reproducibility, controllability, and desired cultivation process performance.

The tool shown in Figure 3 provides a plot of a curve resulting from the gray box model which fits to the fed-batch data illustrated by blue squares on the right side of Figure 3. The blue squares represent the cumulative substrate values acquired from fed-batch *E. coli* cultivation with feeding carried out in portions to a 1 L bioreactor. The total fed substrate mass was 400 g and the cultivation took approximately 12 h after the inoculation. Estimated parameter values were $k_{01}$ = 1.170474, $k_{02}$ = 0.540259, $k_{03}$ = 0.583979, $k_{04}$ = −1.170474, $k_{05}$ = 0, $k_{11}$ = 0, $k_{12}$ = 48.279174, $k_{13}$ = −71.943295, $k_{14}$ = −485.412949 and $k_{15}$ = −2.758384. The calculation took 6244 ms and the relative entropy value was −0.3256.

As can be seen in the left of Figure 3, there are multiple options and alternatives available to a user. These are described, from the top to bottom, below. The first option is to choose an induction time, which determines the intersection point of the two models used in this work. The user can test various time intersections and choose the one that best suits their needs. Sometimes this feature helped the authors to both analyze the client’s data profiles and identify when the true induction activity happened. The initial glucose concentration in a bioreactor defines the expected glucose concentration which must be consumed by bacteria prior to the start of feeding. Therefore, this amount is deducted from the cumulative feeding profile’s start, and the exact time when the feed should be started is recalculated. The start of a feed should be slightly earlier than the time the glucose in cultivation media is exhausted. The glucose reserve option allows starting the feed sooner. The total effect of these latter options result in a feeding start at 4.38 h (bottom of Figure 3).

Next, there is an option, no matter whether limitation is applied to the feeding profile or not, to extend or extrapolate the existing feeding rate time profile compared to the one acquired originally. Otherwise, when no extra hours are defined, the limited total cumulative substrate weight would exactly match the final value of the cumulative substrate value of the original cultivation performed at the maximum feed rate. The first option establishes a linear decrease at the hourly percentage rate, while the second one extrapolates the gray box model by a time variable’s progress according to the exponential model, which already exists inside of the gray box.

The weight of a bottle which contains the substrate solution, if defined, would split the whole feeding profile into multiple interim profiles so that bottles can be replaced during a feed. The developed feeding software also encapsulates a feeding controller, which controls Masterflex pumps and reads the feeding substrate solution weight from weighing scales (e.g., Mettler Toledo, Kern, Axis, or controller with load cells attached, etc.). This allows the user to not only qualitatively change the composition of the feeding solution, but also makes it possible to use smaller, more economical weighing solutions for the cumulative glucose profiles that exceed the maximum measurement range of the weighing mechanism.

**Figure 4:**
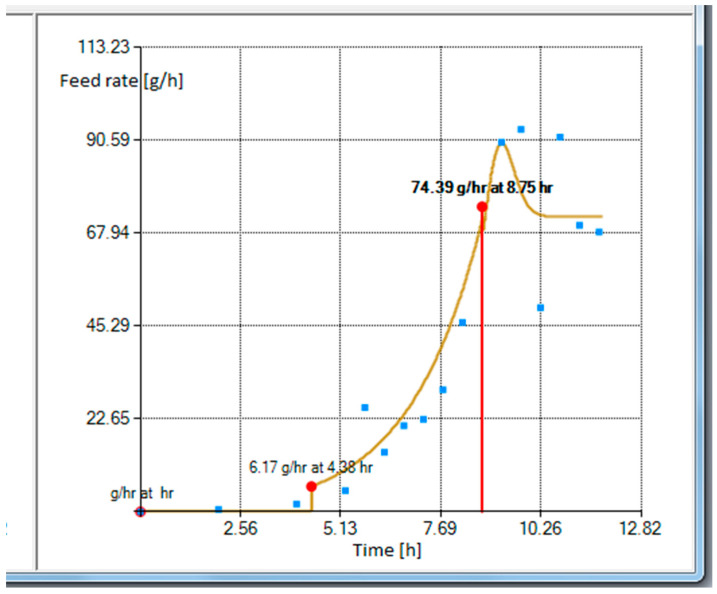
Feeding rate profile view from the right side of the operator dialog screen.

The last option includes an actual limitation tool for the feed rate limitation. This is a key feature of the gray box model-based design allowing the avoidance of changes in metabolic pathways during the cultivation process and tracking the desired glucose consumption trajectory. Scenario 1 contains a single limiting percentage factor for the whole cultivation, and Scenario 2 has two separate limiting percentage factors—the first one for the model prior to the intersection of the profile curves and the other one for the remaining time after the intersection moment. Figure 4 shows the substrate feeding rate profile associated with the cumulative substrate feeding solution in Figure 3.

**Figure 5:**
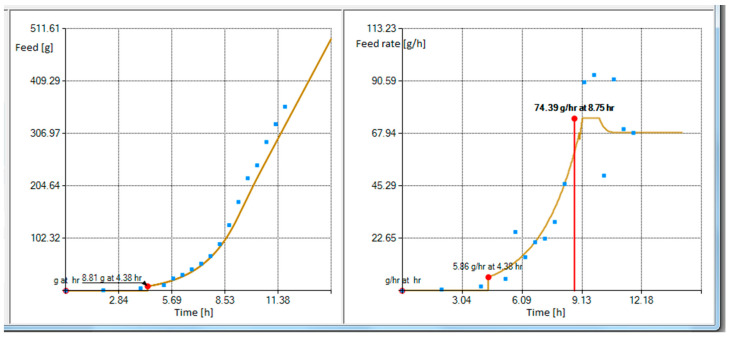
Substrate feeding rate profiles limited to 95%, with the maximum feed rate set to 80% and feeding profile extrapolated, using gray box model, by 2 h.

In Figure 4 the feeding starts at the feeding rate of 6.17 g/h. However, when the profile is limited to 95%, the maximum feed rate is set to 80% and the feeding profile is extrapolated by 2 h, the controller would then start at feeding rate of 5.86 g/h (see Figure 5).

Depending on the product to be maximized, the strain cultivation capabilities, and process duration trade-offs, different limitation alternatives should be designed. A general recommendation is to start with at least three parallel cultivations prior to proceeding with any scale-up. Depending on the output, the gradient direction on what to change next will become evident.

The substrate feeding profile design software tool has a separate dialog for scaling up or scaling down procedure, where the profiles can be scaled relying on the ratio between the initial volumes in a bioreactor vessel. This procedure is also determined by the same gray box model and its optimization using ME. An efficient scale-down procedure has to create conditions that will be representative for the conditions occurring at a large scale.

In conclusion, the gray box model and convex ME optimization both help to design the profile and allow the design of the scale-up, scale-down and prediction strategies in the profile, which is essential for the adaptive control system of bioprocesses.

## 4. Experimental Verification of the Gray Box Model Approach

Three types of verifications are provided by this paper’s gray box model approach. Firstly, OD curves were compared between multiple cultivation processes that had same substrate feeding profile applied. Such a test verifies the reproducibility of the approach and, thus, also its controllability, as defined by the Pareto principle earlier in this paper. Secondly, gray box model fitting to the third party [54] 30-min dataset showed the good descriptive property of this model when decompressing data for nine-second intervals and evaluating its MAPE. Thirdly, this work’s numerical convex approach was validated by analyzing its sensitivity to different combinations of initial computational conditions. The rationality of choosing relative entropy lies in the fact that the proposed convex routine is not sensitive to the initial parameter values, chosen for its iterative procedure, and the resulting MAPE of the estimated model parameters provides an acceptable fit. One of the problems with nonlinear systems is that the best optimization approach can be found for the specific problem and context, yet when initial computational conditions or the model parameters change this approach may no longer be efficient. In this study, a resulting MAPE sensitivity to the artificial choice of different extreme initial parameter seed values demonstrates why this convex approach, when using relative entropy, is a rational practical choice.

### 4.1. Verification of Reproducibility and Controlability

**Figure 6:**
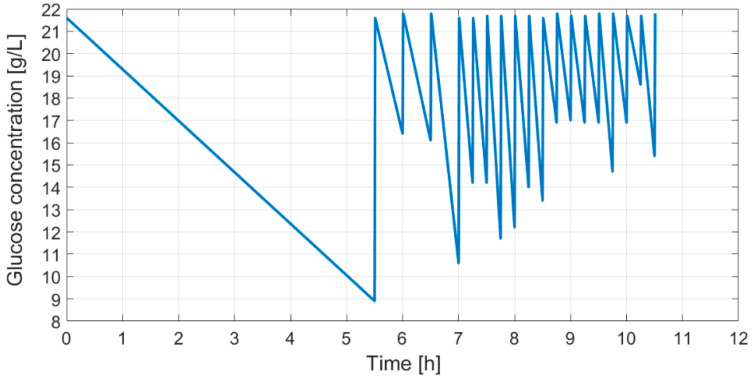
Glucose concentration profile in the bioreactor from the start of inoculation.

As shown in Figure 6, a fed-batch cultivation with dosed glucose feeding provides a profile of glucose concentration in the bioreactor. This profile, as described earlier, with substrate feeding carried out in portions, provides information on the cumulative profile representing the maximal oxidative capacity of cells.

**Figure 7:**
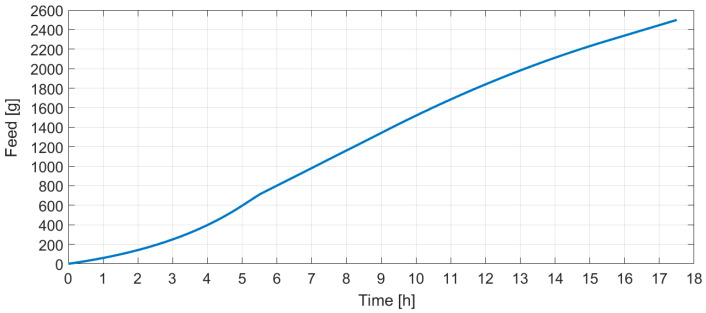
Limiting substrate feeding profile.

After the discussed application of the gray box model, the fed-batch cultivation with feeding carried out in portions produces the cumulative glucose feeding profile (Figure 7) that was tested with three cultivations.

**Figure 8:**
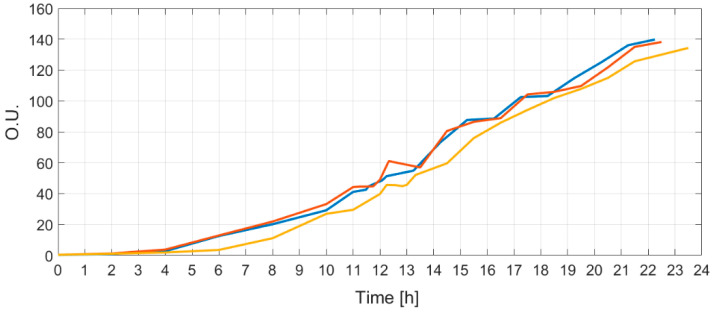
The resulting optical density curves (in optical units) of all three limited growth cultivations.

Each cultivation had an identical initial bioreactor volume, initial glucose concentration, medium OD, and *E. coli* strain inoculated at the beginning of the process. All three curves of optical densities are shown in Figure 8.

Identical substrate feeding solutions were prepared for all three cultivations and their bioreactors were equipped with identical means to perform the biosynthesis. The resulting optical density curves show that there is a reproducibility, as all optical curves have similarity and controllability since the final OD at the end of the limited growth process matched.

### 4.2. Verification of Decompression Property Using the Gray Box Model

For illustrative purposes, the third party’s [54] first cultivation dataset was used to compare descriptive properties of the gray box model between 30-min and nine-second intervals. Both MAPE coefficients showed a consistent match in Table 1.

The gray box model parameters were estimated from the dataset consisting of 30-min data. Then, those parameters were used to decompress the profile to nine-second data and compare it with the dataset provided in [54]. The resulting gray box model parameter values, estimated from the 30-min dataset, were $k_{01}$ = 4.967242, $k_{02}$ = 0.519979, $k_{03}$ = −0.013456, $k_{04}$ = −4.967242, $k_{05}$ = 0, $k_{11}$ = −0.201855, $k_{12}$ = 1.945063, $k_{13}$ = −114.651975, $k_{14}$ = −385.93917 and $k_{15}$ = −0.13398.

The next task is to assess the sensitivity of the numerical procedure described in this work to the choice of initial seed values for the iterative procedure.

### 4.3. Verification of Sensitivity to Initial Seed Values of the Gray Box Model Parameters

Table 2 shows estimates for all gray box parameter values when different seeds are used for the values of the initial parameters $k_{02}$, $k_{12},$ and $k_{15}$ in numerical iterative routines. The execution time was measured on a single process thread using a 2.5 GHz processor.

Both of the parameters $k_{02}$ and $k_{12}$ correspond to the specific growth rate, so their chosen variation is relatively extreme compared to the expected specific growth rates of *E. coli* strains, which are positive and are usually less than 1. The parameter $k_{15}$ corresponds to the acceleration component of the specific growth rate, and normally could be kept 0 at its seed. However, an extreme value of −3 (in Test 4) was used for this parameter’s seed to check that convex routines converge to the semi-globally optimal answer and the MAPE is still acceptable. By monitoring MAPE values for different combinations of initial seeds to the parameter values, there is evidence that the numerical program is not sensitive to the seed values, which provides a significant practical benefit. It is clear that neither optimization execution duration nor the value of relative entropy are dependent on the set of the seeds. The different combinations of local extrema in parameter solution space confirms the property of the undetermined model to have multiple local extrema which are very close to the global optimum, thus providing semi-globally optimal solutions.

In conclusion, the MAPE values do not increase more than 0.32% (Table 2) at different initial conditions, while process dynamics change (OD is proportional to biomass) by approximately 300 times (Figure 8). All this shows that the proposed approach is practically acceptable and produces satisfactory estimation results.

## 5. Discussion

In this study, a generic bioprocess gray box modeling approach is presented that uses entropy maximization to derive an optimization criterion for planning and/or prediction of the feeding solution profile. The proposed design procedure has benefits for the improvement of existing industrial feed-forward and adaptive feedback control systems. Because of the proposed procedure, process controllability is gained, ensuring good control qualities of *pO*_2_, PH, temperature, and substrate feeding parameters, the signals of which all provide information for the implementation of gain scheduling algorithms in such control systems. To find the gray box model parameters in both of the feedback control scenarios, the cumulative glucose volume is introduced as uncertainty into the convex optimization task. A description of nuisance time is provided, which explains how to efficiently arrive at the multivariate solution. Additionally, experimental data analysis was conducted with practical illustration of bioprocess reproducibility and controllability, assessment of goodness of fit to a dataset acquired from a third party, and sensitivity analysis of the numerical routines to the seed values of initial parameters, which shows one more practical benefit of the presented approach.

In the scenario of a regular open-loop biotechnological system, in order to evaluate the specific growth rate profile, the first fed-batch cultivation process with feeding carried out in portions is performed. The volume of the substrate fed during fed-batch steps and observations of glucose concentration in the bioreactor media all help to evaluate the substrate consumption. After the gray box model parameters are identified through ME, the procedures to realize industrial cultivation processes under growth-limited conditions are described, with emphasis on applying different limitation options and alternatives. Glucose concentration measurements become unnecessary during these subsequent cultivations to identify the best run time profile, which corresponds to the desired trajectories of process variables (the specific growth rate and the total biomass). In the adaptive control system, the authors’ approach serves as a short-term forecasting tool, which helps to both design the feed rate time profiles and predict the induction time in the recombinant *E. coli* cultivation processes.

## Figures and Tables

**Table 1:** Gray box model 30-minute model verification with nine-second data.

Dataset	MAPE (%)	Samples Count
30-min	0.542	27
9 s	0.274	5200

**Table 2:** Comparison between five tests with different seeds for parameters $k_{02}$, $k_{12}$, and $k_{15}$.

Optimization Property	Original Seeds: $k_{02}=0;$ $k_{12}=0;$ $k_{15}=0$	Test 1 Seeds: $k_{02}=3;$ $k_{12}=-3;$ $k_{15}=0$	Test 2 Seeds: $k_{02}=-3;$ $k_{12}=3;$ $k_{15}=0$	Test 3 Seeds: $k_{02}=-3;$ $k_{12}=-3;$ $k_{15}=0$	Test 4 Seeds: $k_{02}=-3;$ $k_{12}=-3;$ $k_{15}=-3$
MAPE (%)	0.274	0.312	0.286	0.275	0.313
Execution time (ms)	888	451	482	1395	500
Relative entropy	−0.0001415	−0.0001746	−0.0001401	−0.0001457	−0.0001764
$k_{01}$	4.967242	4.970157	4.964	4.967	4.970163
$k_{02}$	0.519979	0.519899	0.52	0.52	0.519899
$k_{03}$	−0.013456	−0.011921	−0.017	−0.013	−0.011914
$k_{04}$	−4.967242	−4.970157	−4.964	−4.967	−4.970163
$k_{05}$	0	0	0	0	0
$k_{11}$	−0.201855	−0.128734	−0.214	−0.182	−0.152797
$k_{12}$	1.945063	2.046178	1.932	1.969	2.006798
$k_{13}$	−114.651975	−115.72494	−114.414	−115.086	−115.0439
$k_{14}$	−385.93917	−400.158251	−382.82	−391.63	−391.249
$k_{15}$	−0.13398	−0.140025	−0.133	−0.135	−0.137536
